# Integrating the ecophysiology and biochemical stress indicators into the paradigm of mangrove ecology and a rehabilitation blueprint

**DOI:** 10.1371/journal.pone.0202227

**Published:** 2018-08-13

**Authors:** Abner Barnuevo, Takashi Asaeda

**Affiliations:** 1 Graduate School of Science and Engineering, Saitama University, Sakura-ku, Saitama, Japan; 2 KP Center for Mangrove Research, KGroup Philippines, Inc., iMEZ Bldg., MEZ2, Pueblo Verde, Basak, Lapulapu City, Cebu, Philippines; 3 Department of Environmental Science, Saitama University, Sakura-ku, Saitama, Japan; Universitat de Barcelona, SPAIN

## Abstract

The continuous degradation of mangrove habitats has encouraged governments and multi-lateral agencies to undertake rehabilitation initiatives to foster the recovery and biodiversity of these areas. However, some rehabilitation initiatives suffer high mortality because of incorrect species-site matching and failure to recognize the ecophysiology of mangrove species. This study investigated the effects of salinity, water depth and inundation on the growth, biochemical stress responses, and ecophysiology of *Rhizophora stylosa* in greenhouse conditions. Propagules were cultured in aquarium tanks and irrigated with low (0 ppt), moderate (20 ppt), and high (35 ppt) salinity treatments. In the first setup, the seedlings were cultured in aquarium tanks and arranged on the top of a platform at different elevations, subjecting the seedlings to flooding with low-water (3–5 cm), mid-water (10–13 cm) and high-water (30–33 cm) levels for ten months. In another setup, the seedlings were cultured for 15 months at the low-water level and subjected to inundation hydroperiods: semi-diurnal, diurnal and permanent inundation for one week. These microcosms simulated emerged and submerged conditions, mimicking intertidal inundation that seedlings would experience. The results showed that salinity significantly affected the early development of the cultured seedlings with higher growth rates and biomass at low and moderate salinity than those at high salinity. Levels of reactive oxygen species (ROS) and antioxidant activities (AOX) were significantly lower in the emerged condition than those in an inundated condition. Inundation imposed a higher-degree of stress than that of the salinity effect, with prolonged inundation caused sublethal damage (chlorotic leaves). Furthermore, inundation caused the reduction of photosynthetic pigments and fluorescence, dependent on salinity. Extrapolating the ecophysiology of *R*. *stylosa*, this species had low tolerance to inundation stress (high ROS and AOX, reduced pigments). Translating this low tolerance to field conditions, in the frequently inundated areas (i.e., seafront mangrove fringes) that are subjected to longer inundation at spring tides, this species may suffer from oxidative stress, stunted growth and consequently low survival.

## Introduction

Human exploitation and conversion of natural ecosystems are causing widespread habitat loss and degradation, which translate into a loss and decline in biodiversity and ecosystem services [1]. Coastal ecosystems have received considerable pressure stemming from increasing population density and continuing economic development, with approximately 44% of the world population living within 100 km of the coast [2]. Mangroves are one of the valuable habitats that have suffered a global decimation estimated at 20% from 1980 to 2005 [3], with the highest losses in southeast Asia, primarily due to agriculture and aquaculture conversion [4, 5]. Rehabilitation programs have received significant interest as a tool to restore damaged habitats, however, the results include stories of mixed successes and failures [6, 7]. These efforts were often unsuccessful, because of the high mortality of the seedlings due to the failure to recognize the species-specific environmental tolerances and thresholds [5, 8, 9].

Located at the interface between the land and sea, the survival of mangrove seedlings are under the continuous influence of different environmental drivers specifically salinity and tidal inundation. Tidal inundation is considered to have an important role in the paradigm of mangrove establishment and distribution [10]. However, Friess [11] cautioned that research on tidal inundation and species distribution as previously described by Watson [12] must acknowledge that vegetation-inundation linkages are not universally applicable and that species distribution is multifactorial. On the other hand, the effects of salinity has been extensively studied [12–14], but earlier studies focused only on the growth rate and biomass production. A gap in the knowledge remains linking the mangrove ecophysiology and biochemical stress responses with the environmental drivers.

The combined effects of different abiotic factors disrupt the physiological homeostasis of mangroves and consequently cause the induction of reactive oxygen species (ROS) resulting in oxidative stress and eventual mangrove mortality when conditions remain unfavorable. ROS are higly reactive oxygen derivatives that are formed as a by-product of metabolism. However, during times of stressful conditions, ROS are significantly generated resulting to damage in cell structures. As a response to counter the adverse effects of the increase in ROS, mangroves have developed natural defense system including the activation of antioxidant enzymes (AOX) to scavenge the ROS, and a range of ecophysiological responses in metabolism and biomass partitioning [15–18]. Understanding the ecology and adaptability of mangroves to the interplay of abiotic stressors requires an interdisciplinary knowledge of botany, physiology, geography, and molecular and genetic sciences [11]. However, the ecophysiology and species-specific niche-width are not well established. Knowledge of the levels of biochemical stress to abiotic drivers can serve as a tool in elucidating the ecophysiology of a species, providing vital information for selecting optimum conditions and suitable areas for science-based mangrove rehabilitation programs. This study investigated the biochemical stress responses and ecophysiology of *Rhizophora stylosa*, a commonly selected species for rehabilitations, to different levels of salinity and inundation period in greenhouse conditions.

## Methods

### Studied species and propagule collection

Mangrove rehabilitation programs have been conducted extensively to restore mangrove cover and habitat functionality. Specifically, in southeast Asia, rehabilitation programs are conducted through the planting of mangrove propagules, with *Rhizophora* species frequently used because of the availability of the mature propagules throughout the year. Such propagules are convenient to collect and can be directly planted without the requirement of a nursery culture period. Thus, species of *Rhizophora* are popularly chosen for many rehabilitation initiatives.

Mature propagules of *Rhizophora stylosa* were collected from Olango Island, Lapu-lapu City, Cebu, in the central Philippines and brought to Saitama University, Japan, for greenhouse culture. The island where the species was sourced was declared as the first Ramsar site of the Philippines. The mangrove forest is classified as fringing forest with water salinity up to 36 ppt and no freshwater input from a river. The island is surrounded by broad sandy beaches and rocky shorelines, inshore flats, sea grass beds, reefs, mangrove forests and mudflats [6, 7]. Mangrove rehabilitation efforts have been conducted for the last 30 years using the species *R*. *stylosa* to revert the habitat loss initiated by the government and varous non-government organizations.

### Greenhouse culture and effects of sainity and water depth

After two weeks of acclimatization, the propagules were individually planted in seedling bags filled with mixed washed river sand and vermiculite at a 4:1 ratio. The seedlings were maintained inside aquarium tanks (120 x 45 x 45 cm) irrigated with different salinities: low (0 ppt), moderate (20 ppt) and high (35 ppt), prepared from Instant Ocean, Aquarium Systems [19–21]. In the tanks, the seedlings were arranged on the top of a platform made from a pile of bricks designed to create an elevation gradient, and the seedlings were irrigated with low water (LW, 3–5 cm), mid-water (MW, 10–13 cm) and high water (HW, 30–33 cm). In LW, only the soil pot is flooded; in MW, 50% of the planted propagule is flooded; and in HW, 95% of the planted propagule is flooded. Throughout the culture period, the leaves of the seedlings were above the water level and this setup was referred to as the emerged condition in the data analyses and interpretation. Greenhouse conditions were maintained with a 12 h photoperiod and at 27±5°C. The development and unfurling of the first leaves were monitored, and the average height and relative growth rate (RGR) were measured monthly. After 10 months, the seedlings were harvested, and leaf tissue samples were immediately assayed for reactive oxygen species (ROS), specifically hydrogen peroxide, antioxidants (catalase, ascorbate peroxidase, guaiacol peroxidase) and pigments (chlorophyll a and b, carotenoids) as detailed below. The above- and belowground tissues were partitioned, and the biomass was determined by oven drying of the harvested samples at 80°C for 72 h until constant dry weight was obtained.

### Effects of salinity and inundation period

A second set of seedlings was cultured in tanks irrigated with the LW level (3–5 cm) and with the three different salinity treatments similar to the first experiment. After 15 months, the cultured seedlings were then subjected to inundation hydroperiods simulating the tidal cycle as semi-diurnal inundation (SDI), diurnal inundation (DI) and permanent submersion (PS) for one week. Mangrove areas are frequently inundated twice a day during spring tides and occasionally once a day during neap tides. Thus, the use of the semi-diurnal and diurnal inundation to mimicked the tidal inundation cycle. For the SDI, the seedlings were inundated twice a day for three hours per inundation exposure and with three hours of drained period in between inundations; for the DI, the seedlings were inundated for 6 h per day; and for the PS, the seedlings were permanently inundated for 24 h. For SDI and DI, when in the emerged condition, the water inside the experimental tank was drained and siphoned to another tank with a pump until only 3–5 cm of water depth remained (similar to the culture condition). When in the inundated condition (SDI, DI, PS), the topmost pair of leaves was 10–15 cm below the water surface. The seedlings were observed daily for impacts, specifically on the leaves. The number of seedlings and the experimental tanks included three replicates for each of the inundation periods, and at the end of the experiment, leaf tissue samples were collected and assayed as detailed below.

### Extraction and analyses of pigments, fluorescence, H_2_0_2_ and enzymes

The leaf pigments were extracted with N,N-dimethylformamide for 24 h and measured with a spectrophotometer. The chlorophyll a and b and carotenoid concentrations were calculated based on Wellburn [22]. Fluorescence was measured using a chlorophyll fluorescence imaging technique (FC 1000-H; Photon Systems Instruments, Czech Republic) with auto image segmentation. Leaf samples were dark-adapted for 20 min, and the maximum quantum efficiency of photosystem II photochemistry (Fv/Fm) was calculated following DeEll and Toivonen [23].

Assays for hydrogen peroxide (H_2_O_2)_, catalase activity (CAT), ascorbate peroxidase activity (APX) and peroxidase activity (POD) were performed by grinding the fresh leaf samples (300–500 mg) using a mortar and pestle and liquid nitrogen with ice-cold 50 mM phosphate buffer (pH 6.0) and polyvinylpyrrolidone. The extracts were centrifuged at 3000 × g and 4°C for 15 minutes, and the supernatant was separated for subsequent assays. The H_2_O_2_ was determined based on Jana and Choudhuri [24]. An aliquot of 750 μL was mixed with 2.5 mL of 0.1% titanium sulphate in 20% H_2_SO_4_ (v/v) and the mixture was centrifuged at 5000×g for 15 minutes at 20 ^o^C. The intensity of the resulting yellow color was measured at 410 nm using a spectrophotometer. The CAT activity was measured according to Aebi [25]. The reaction mixture was prepared with 100 μL of 10 mM H_2_O_2_ and 2.00 mL of 100 mM potassium phosphate buffer (pH 7.0). An aliquot of 500 μL of the enzyme extract was added to the reaction mixture and the absorbance reduction was measured with a spectrophotometer at 240 nm for every 10 s for three minutes. The APX activity was measured according to Nakano and Asada [26]. The reaction mixture contained 100 μL of enzyme extract, 200 μL of 0.5 mM ascorbic acid in 50 mM potassium phosphate buffer (pH 7.0) and 2.0 mL of 50 mM potassium phosphate buffer (pH 7.0). The reaction was started by adding 60 μL of 1 mM H_2_O_2_ and the reduction in absorbance was measured with a spectrophotometer at 290 nm every 10 s for three minutes. The POD activity was measured based on MacAdam, Nelson [27]. The reaction mixture contained 3.0 mL of 50 mM potassium phosphate buffer (pH 6.0), 40 μL of 30 mM H_2_O_2_ and 50 μL of 0.2 M guaiacol. The reaction was started by the addition of 100 μL of enzyme extract, and the increase in absorbance was measured at 420 nm every 10 seconds for 3 minutes.

### Data analyses and statistics

Statistical analyses were conducted in XLSTAT Premium. The growth and morphological measurements and the biochemical stress responses of cultured seedlings to water level, salinity and inundation period were evaluated for normal distributions and for homogeneity of the variances. Two-way analysis of variance (2-way ANOVA) was performed to examine the effects of salinity, water depth and inundation period on all the response variables (growth, biomass, pigments, ROS, AOX) at a significance level of p<0.05. The differences (induction or reduction) of the response variables of the cultured seedlings in emerged (reference) and inundated conditions were evaluated to determine the most stressful conditions and identify the most influential variable affecting the ecophysiology of *R*. *stylosa*.

## Results

### Influence of salinity and water depth on seedling establishment, growth and biomass

**Fig 1:**
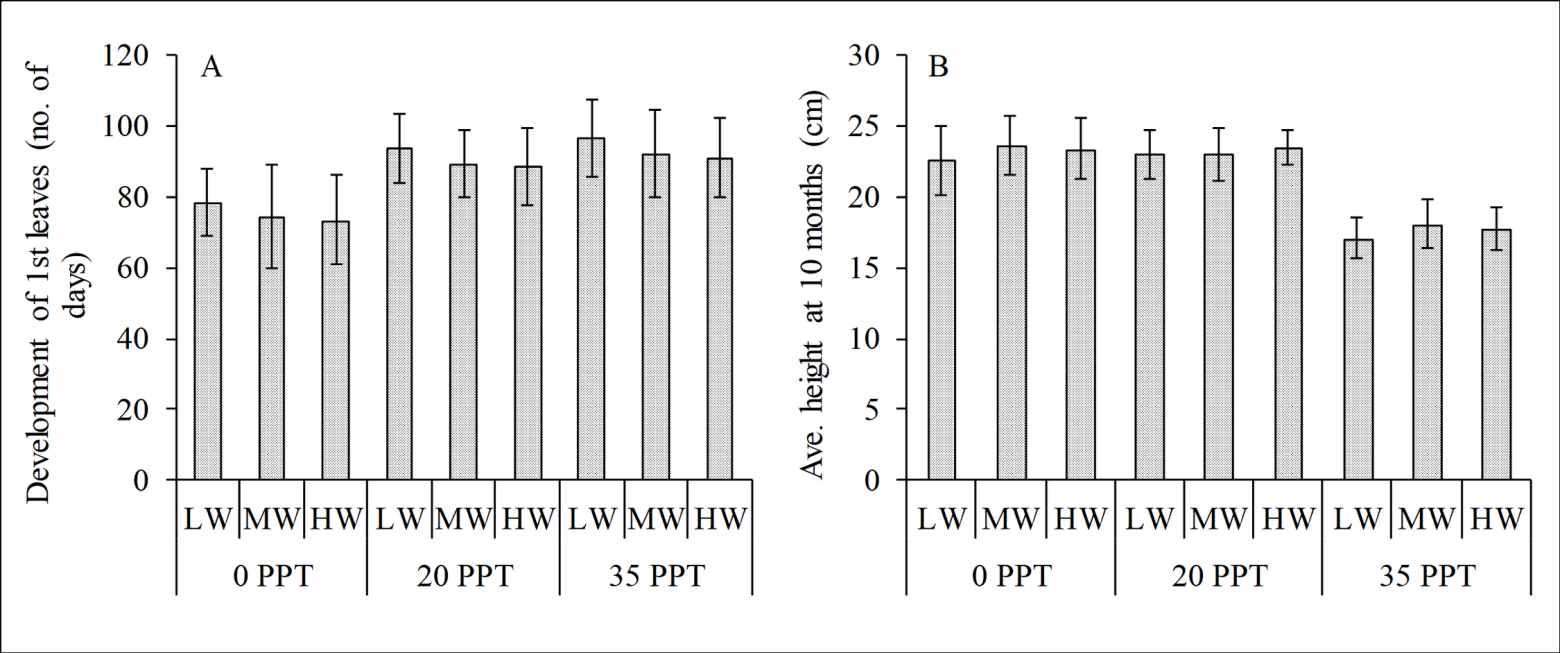
Initial development and growth rate of cultured *Rhizophora stylosa*. (A) Development of the first leaves (no. of days) and (B) average height (cm) at 10 months in low (0–3 ppt), moderate (17–20 ppt) and high (33–36 ppt) salinity treatments. In each salinity treatment, the seedlings were irrigated with low (LW), mid (MW) and high water (HW) levels.

Salinity influenced the initial establishment and growth of the cultured *R*. *stylosa* propagules. The first pair of leaves unfurled relatively faster for the seedlings cultured in low salinity than for those in moderate and high salinity treatments (Fig 1A). Low and moderate salinity provided favorable conditions for the growth rate of cultured seedlings over 10 months (Fig 1B), whereas the water depth and the interaction of salinity and water depth had no effect based on the 2-way ANOVA (Table 1). The optimum growth rate was observed in the moderate and low salinity cultured seedlings with average heights of 23.2±1.88 and 23.4±1.59 cm, respectively, whereas the high salinity cultured seedlings had an average height of 17.67±1.56 cm. The seedlings cultured in high salinity showed retarded growth with an RGR of 1.77±0.16 cm/month. Although the water level per salinity treatment showed a slight difference in growth rate values and average heights, the differences were not significant.

**Table 1:** Two-way ANOVA showing the effects of salinity and water depth on the development and growth of *R*. *stylosa* seedlings in the emerged condition cultured for 10 months.

Response	Factor	*F*	*P*	R^2^
Average height	Salinity	26.353	< 0.0001	0.763
	Water depth	0.475	0.630	
	Salinity x water depth	0.022	0.999	
Leaf area	Salinity	100.255	< 0.0001	0.928
	Water depth	0.306	0.740	
	Salinity x water depth	2.898	0.054	
Stem diameter	Salinity	2.048	0.160	0.311
	Water depth	0.435	0.654	
	Salinity x water depth	0.586	0.677	
Leaf biomass	Salinity	22.915	< 0.0001	0.743
	Water depth	0.578	0.572	
	Salinity x water depth	0.129	0.970	
Stem biomass	Salinity	330.732	< 0.0001	0.976
	Water depth	3.420	0.056	
	Salinity x water depth	1.019	0.425	
Root biomass (or BGB)	Salinity	134.542	< 0.0001	0.941
	Water depth	1.336	0.289	
	Salinity x water depth	0.063	0.992	
Shoot biomass (or AGB)	Salinity	188.777	< 0.0001	0.960
	Water depth	5.564	0.014	
	Salinity x water depth	0.324	0.858	

**Fig 2:**
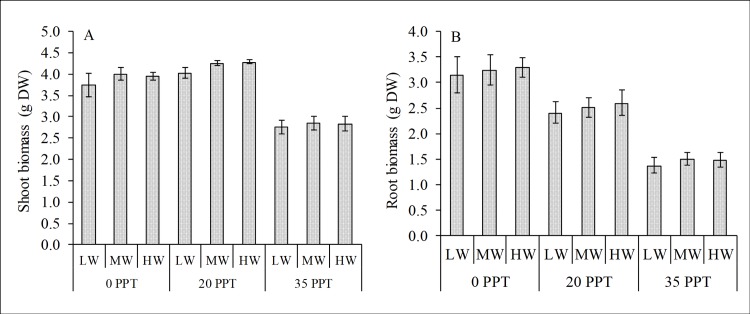
Biomass production of cultured *R*. *stylosa* harvested after a 10-month culture period. (A) Aboveground biomass or shoot biomass and B) belowground biomass or root biomass in three salinity treatments with three water depths per treatment.

Salinity also significantly influenced the biomass of the cultured seedlings; whereas the effects of water depth and the interaction of salinity and water depth were not significant (Table 1). The seedlings cultured in moderate salinity had the highest AGB with 4.19±0.08 g DW, followed by those cultured in low salinity with 3.90±0.17 g DW, whereas the AGB of seedlings cultured in high salinity was 2.82±0.16 g DW (Fig 2A). For root biomass or BGB, seedlings cultured in low salinity had the highest (3.23±0.28 g DW), followed by seedlings in moderate salinity (2.52±0.22 g DW), whereas seedlings in high salinity had the lowest with only 1.46±0.14 g DW (Fig 2B). In terms of biomass allocation and partitioning, the AGB was relatively higher than the BGB as shown by the root-to-shoot ratio (R/S), and the seedlings cultured in low salinity had the highest R/S ratio (0.83%), followed by the seedlings cultured in moderate salinity (0.60%), whereas the high salinity cultured seedlings had the lowest (0.52%).

### Effects of salinity and water depth on pigments and biochemical stress responses

**Fig 3:**
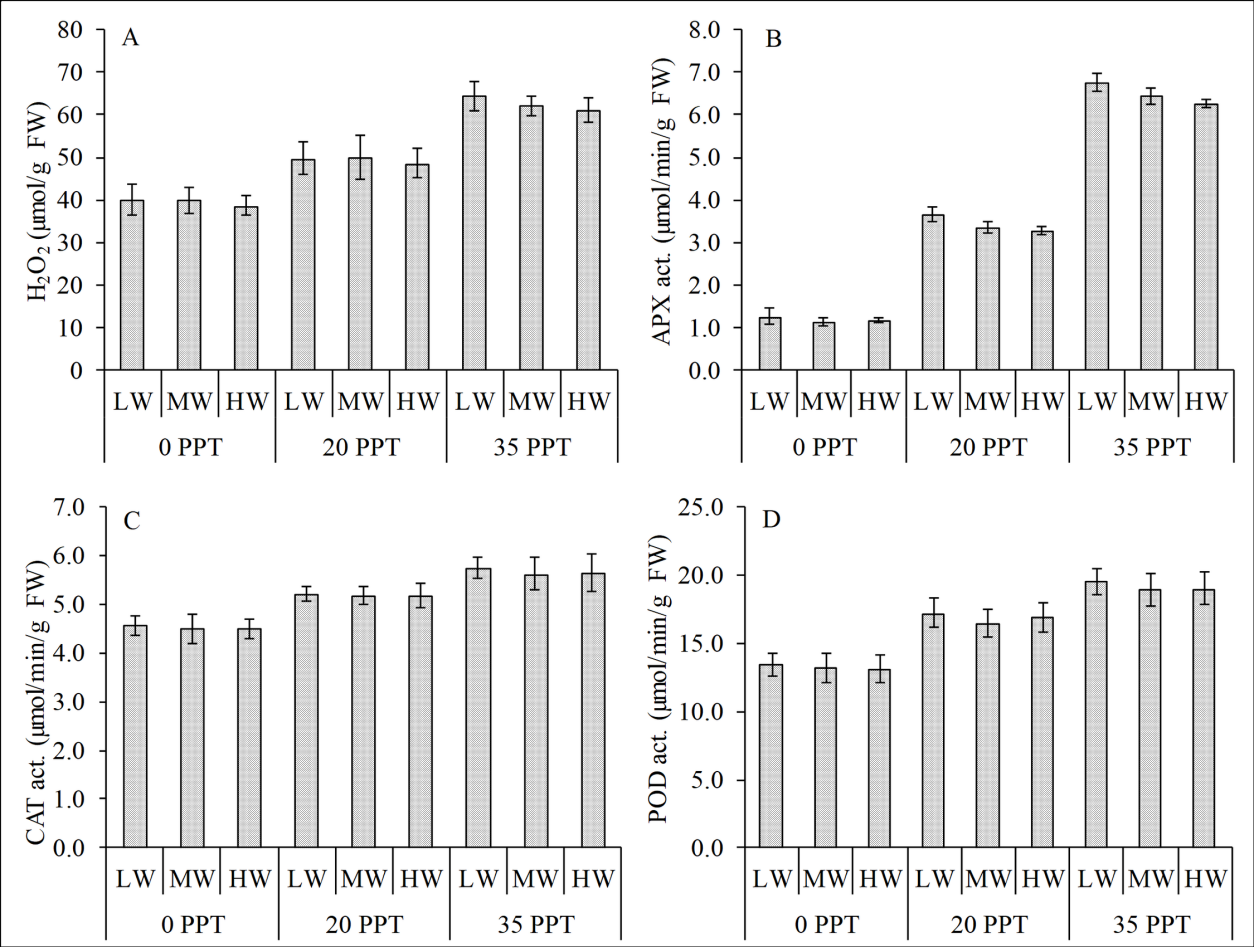
Relationship of reactive oxygen species and antioxidant enzyme activities in the leaves of *R*. *stylosa*. (A) H_2_O_2_ concentration and antioxidant activities of (B) APX, (C) CAT and (D) POD in the emerged condition in three salinity treatments with three water depths per treatment.

In the emerged condition, salinity had a significant effect on the differences in the biochemical stress responses; whereas the effects of water depth and the interaction of salinity and water depth were not significant (Table 2A). The leaf H_2_O_2_ showed a strong variation (R^2^ = 0.917) with increasing salinity (Fig 3A). The seedlings cultured in low salinity had the lowest H_2_O_2_, followed by the seedlings cultured in moderate salinity, whereas the seedlings in high salinity had the highest. Based on the 2-way ANOVA, salinity had significant effect on the variability of H_2_O_2_ production, whereas water depth and the interaction of salinity and water depth had no effect (Table 2A).

**Table 2:** Two-way ANOVA showing the effects of (A) salinity, water depth and (B) salinity and inundation on *R*. *stylosa* seedlings. A and B correspond to emerged and submerged conditions, respectively.

a.) Effects of salinity and water depths					b.) Effects of salinity and inundation				
Response	Factor	*F*	*P*	R^2^	Response	Factor	*F*	*P*	R^2^
H_2_O_2_	Salinity	98.82	<0.0001	0.92	H_2_O_2_	Salinity	7.3	0.005	0.90
	Water depth	0.74	0.491			Inundation	62.2	<0.0001	
	Salinity x water depth	0.12	0.975			Salinity x inundation	4.7	0.009	
APX	Salinity	2979.12	<0.0001	1.00	APX	Salinity	1058.8	<0.0001	1.00
	Water depth	12.74	0.000			Inundation	454.7	<0.0001	
	Salinity x water depth	1.47	0.252			Salinity x inundation	13.5	<0.0001	
CAT	Salinity	42.72	<0.0001	0.84	CAT	Salinity	18.9	<0.0001	0.96
	Water depth	0.64	0.539			Inundation	197.1	<0.0001	
	Salinity x water depth	0.21	0.928			Salinity x inundation	0.1	0.970	
POD	Salinity	73.04	<0.0001	0.90	POD	Salinity	18.4	<0.0001	0.98
	Water depth	0.85	0.444			Inundation	460.1	<0.0001	
	Salinity x water depth	0.07	0.990			Salinity x inundation	1.5	0.234	
Chl a	Salinity	10.12	0.001	0.57	Chl a	Salinity	41.1	<0.0001	0.95
	Water depth	0.73	0.494			Inundation	120.8	<0.0001	
	Salinity x water depth	0.15	0.960			Salinity x inundation	1.4	0.280	
Chl b	Salinity	51.97	<0.0001	0.87	Chl b	Salinity	25.2	<0.0001	0.94
	Water depth	0.79	0.468			Inundation	96.4	<0.0001	
	Salinity x water depth	0.09	0.983			Salinity x inundation	1.0	0.455	
Car	Salinity	34.68	<0.0001	0.80	Car	Salinity	48.1	<0.0001	0.89
	Water depth	0.10	0.902			Inundation	58.0	<0.0001	
	Salinity x water depth	0.15	0.960			Salinity x inundation	1.0	0.450	
Fv/Fm	Salinity	0.22	0.802	0.03	Fv/Fm	Salinity	3.4	0.056	0.76
	Water depth	0.01	0.991			Inundation	23.4	<0.0001	
	Salinity x water depth	0.00	1.000			Salinity x inundation	0.9	0.461	

Salinity had a significant effect on the activities of APX, CAT and POD, whereas the water depth and the interaction of salinity and water depth had no effect. Highest enzyme activities were in plants cultured high salinity while the lowest were those cultured in the low salinity (Fig 3B, 3C and 3D). The activites of the enzymes increased as the H_2_O_2_ increased; signifying its role as the natural defense system to lessen the adverse effect of increasing H_2_O_2_.

**Fig 4:**
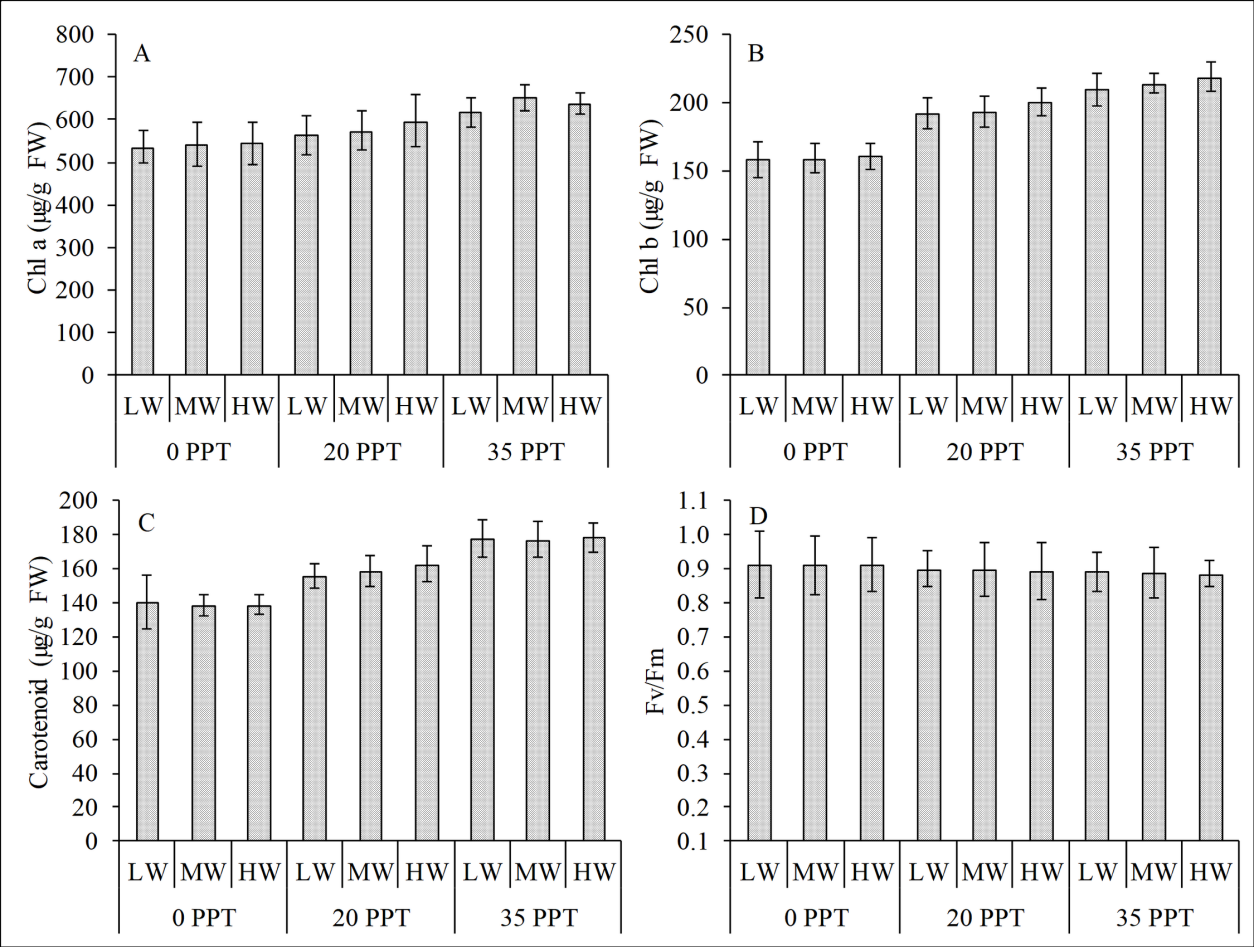
Pigments, carotenoids and Fv/Fm ratio in the emerged condition. (A) Chl a, (B) chl b, (C) carotenoids and (D) Fv/Fm ratio in the leaves of *R*. *stylosa* cultured for 10 months in three salinity treatments with three water depths per salinity treatment.

Salinity also significantly affected the variability of Chl a, Chl b and carotenoids, whereas the effects of water depth and the interaction of salinity and water depth were not significant (Table 2A). Both pigments and carotenoids were positively correlated with the increasing salinity (Fig 4A, 4B and 4C). By contrast, the Fv/Fm ratio showed showed no significant different in the salinity treatment and water depth (Fig 4D).

### Effects of periodic inundation and submersion on biochemical stress responses

**Fig 5:**
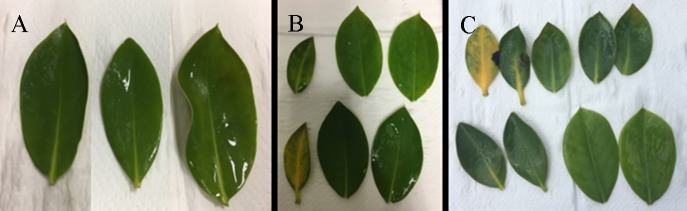
Photos of the harvested leaves of cultured *R*. *stylosa* subjected to prolonged inundation. The leaves of the seedlings in (A) low salinity showed no chlorosis after seven days, whereas the seedlings exposed to (B) moderate salinity showed chlorosis after five days and those exposed to (C) high salinity developed chlorotic leaves after four days.

Periodic inundation (SDI and DI) and prolonged inundation (PS) induced a higher-order magnitude of stress than the effects of salinity (Table 2B). The PS even caused sublethal damage as manifested by the chlorosis of the leaves (Fig 5) in high and moderate salinity treatments. The chlorotic leaves developed faster in high salinity than in moderate salinity, appearing after four days in the high salinity treatment and after five days in the moderate salinity treatment; whereas in low salinity, chlorosis was not observed even at the end of the experimental period.

**Fig 6:**
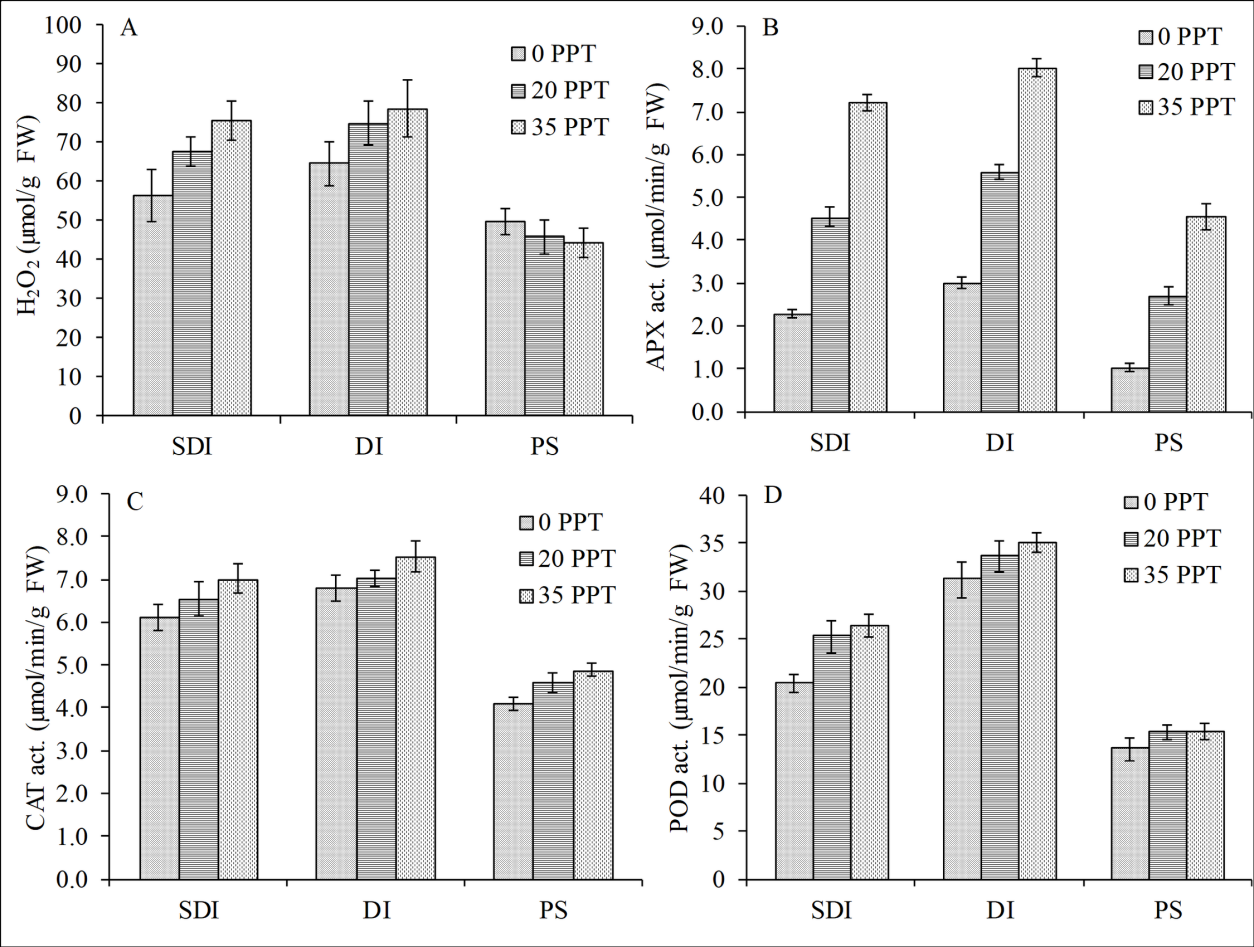
Influence of periodic and prolonged inundation on ROS and antioxidant enzymes. Whereas periodic inundation increased the concentration and activities, prolonged inundation caused reductions in the (A) concentration of H_2_O_2_ and activities of (B) APX, (C) CAT and (D) POD in the leaves of *R*. *stylosa* seedlings cultured for 10 months in three salinity treatments with three water depths per treatment.

The periodic inundation caused a significant increase in the generation of H_2_O_2_ both for SDI and DI, whereas the generation significantly decreased for the PS (Fig 6A). However, the reduction in H_2_O_2_ concentration in the PS condition was due to the sublethal damage manifested by the yellowing of leaves, indicating that the plants could no longer produce the ROS. Based on the 2-way ANOVA, salinity, inundation and the interaction of salinity and inundation had significant effects on the H_2_O_2_ variations (F = 7.30, p<0.05; F = 62.15, p<0.0001; F = 4.73, p<0.05, respectively). Among these variables, inundation was the most influential factor (Table 2B). Similarly, the periodic inundation significantly induced the activities of APX (F = 1,058.79, P<0.0001), CAT (F = 197.15, P<0.0001) and POD (F = 460.06, P<0.0001) (Fig 6B, 6C and 6D). Salinity also significantly affected the variation in activities of these enzymes; however, the effect of inundation was a higher order of magnitude than that of salinity (Table 2B). By contrast, for the PS, all the AOX were significantly reduced relative to the reduction of H_2_O_2_, which was attributed to the sublethal damage of the leaf tissues.

**Fig 7:**
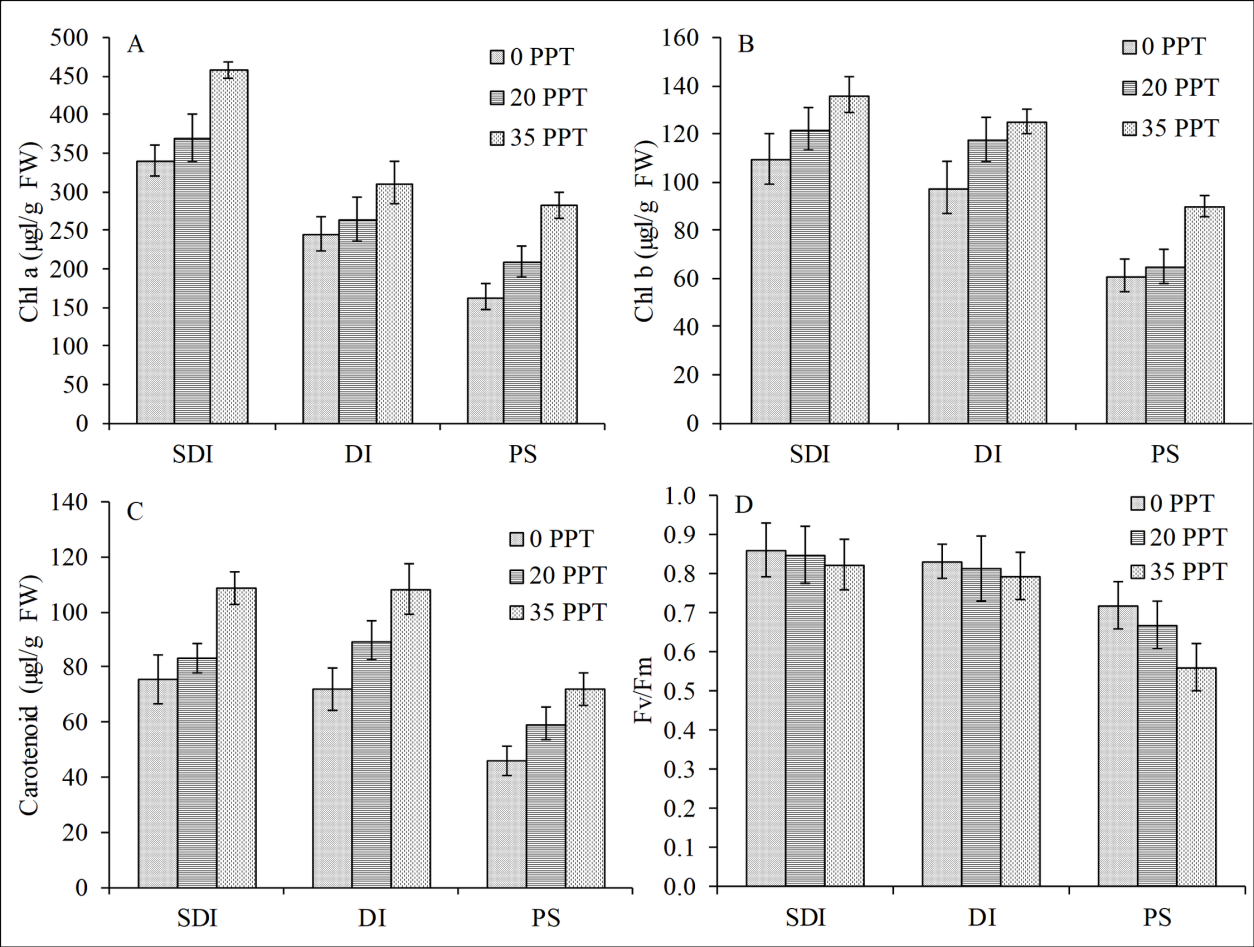
Influence of periodic and prolonged inundation on pigments and fluorescence. Both inundation hydroperiods caused reductions in (A) chl a, (B) chl b, (C) carotenoids and (D) Fv/Fm ratio of *R*. *stylosa* seedlings cultured for 10 months in three salinity treatments with three water depths per treatment.

The pigments (chl a and b), carotenoids and Fv/Fm ratio showed a significant reduction relative to the inundation hydroperiod in all salinity treatments, and the PS condition showed the highest reduction (Fig 7A, 7B, 7C and 7D). For pigments and carotenoids, inundation and salinity both significantly influenced the reduction, but the inundation was the most influential factor (Table 2B). However, for the Fv/Fm ratio, only inundation had a significant effect. As previously stated, this reduction in pigments, carotenoids and Fv/Fm ratio was attributed to the observed morphological damage of the leaf tissues manifested by the yellowing of the leaves and implied that *R*. *stylosa* seedlings had low tolerance to prolonged underwater stress.

## Discussion

### Influence of salinity, water depth and inundation

This study provides a platform for further understanding the tolerances and thresholds of an important mangrove species for rehabilitation, *R*. *stylosa*, to salinity and inundation hydroperiod by integrating its biochemical stress indicators and ecophysiology. The results of the study showed that periodic inundation induced a higher-degree of stress than the effects of salinity, in contrast prolonged inundation even caused sublethal damage as manifested by the chlorosis of the leaves of cultured *R*. *stylosa*. This implies that this species had low tolerance to prolonged underwater stress, as also shown by the significant induction in ROS and AOX and by the reduction of the photosynthetic pigments and Fv/Fm ratio. The magnitude of stress caused by inundation was synergistically influenced by salinity, with higher stress for seedlings cultured in high salinity than for those in low and moderate salinity treatments.

Salinity is one of the primary abiotic drivers in mangrove growth and early development [13, 28], and the salt tolerance mechanisms of mangroves have been the focus of several studies. The seedlings cultured in high salinity showed delayed initial development and lower growth rate and had significantly lower AGB and BGB than those cultured in low and moderate salinity treatments. It has been reported that mangroves exhibit species-specific salinity tolerances. Based on a study by Aziz and Khan [29], the species *Ceriops tagal* has optimum growth at 50% seawater; a study by Jayatissa, Wickramasinghe [30] found that *Sonneratia caseolaris* optimum growth is at low salinity (3–5 ppt); whereas Chen and Ye [31] reported that optimum growth of *Excoecaria agallocha* was below 5 ppt. Although most studies find that seedlings grow best at 25% seawater, high salinity (50 to 75% sea water) or the total lack of salt (i.e., freshwater) also affect growth [32]. This phenomenon is considered an expression of a physiological trait of mangroves that demands salt [33]; however, no studies have attempted to explain the mechanism. Contradictory views remain regarding the relationship between mangroves and salt and whether mangroves are facultative or obligate halophytes [34].

Although the importance of tidal flooding and inundation in mangroves has long been reported [12], substantial gap in the knowledge remains on the species-specific ecophysiological responses to inundation hydroperiod. Many of the contemporary studies on the establishment and early development of mangroves have either ignored the effects of flooding within laboratory settings or have failed to quantify tidal inundation in the field. This study on *R*. *stylosa* seedlings showed that inundation caused a significant induction of ROS and reduction of pigments and carotenoids. Additionally, when underwater, the processes of photosynthetic activity and fluorescence were adversely affected. A study by Hoppe-Speer, Adams [35] on the response of *R*. *mucronata* to salinity and inundation showed that photosynthetic performance and stomatal conductance were lowest in the continuous inundation treatment. Furthermore, plants that were exposed to high salinity and continuous inundation have manifested symptoms of leaf necrosis. A study by Pezeshki, DeLaune [36] on *Avicennia germinans* and *Lagunncularia racemosa* found a significant reduction in total leaf area in response to flooding, and a study by Ye, Tam [37] showed that the relative growth rate of *B*. *gymnorrhiza* decreased with duration of flooding. Similarly, a study by Chen, Wang [16] on seedlings of *K*. *candel* found a reduction of photosynthetic light saturation levels and photosynthesis and intercellular CO_2_ with longer immersion. The submergence study of Mangora, Mtolera [38] on *A*. *marina*, *B*. *gymnorrhiza* and *Heritiera littoralis* seedlings showed that the survival and photosynthetic rates decline with increasing salinity and submergence time.

### Linking the interplay of the abiotic stressors and biochemical responses

Inhabiting the intertidal region, mangroves are continuously exposed to the interplay of abiotic stressors that alters mangrove physiology and consequently triggers the excessive generation of ROS in the mitochondria, chloroplast and peroxisomes [39]. ROS are derivatives of oxygen that are highly reactive and include hydrogen peroxide, hydroxyl radical, superoxide and singlet oxygen. In a biological context, ROS are formed as a natural by-product of the normal metabolism of oxygen and have important roles in cell signaling. However, during times of environmental stress, levels of ROS dramatically increase resulting in significant damage to cellular structures. As a natural response to the increases in ROS, mangroves developed an efficient non-enzymatic and enzymatic antioxidant defense system to counter the deleterious effects of ROS [40]. Increases in levels of ROS result in increased relative abundance of several ROS scavenging enzymes such as CAT, POD and APX, among others [41]. When the ROS production exceeds the scavenging activity of the natural defense mechanism, plants suffer from oxidative stress with consequent effects on physiology, biochemistry, cellular activities and nutrient uptake that result in the reduction of growth and biomass. In this study, periodic inundation and prolonged submersion caused the induction and reduction of ROS in the leaves of *R*. *stylosa*. The stress level is significantly higher in the inundated condtion compared with the emerged condition. Inundation also reduced the photosynthetic capacity and the Fv/Fm ratio, which could be translated into reduction of plant growth and lowering of productivity at the community level.

Other studies show that as a consequence of the stress generated by inundation, the stomata close, subsequently causing the down-regulation of the photosynthetic machinery and leading to the generation of ROS in the chloroplast [42]. A study by Wang, Xiao [43] showed a direct relationship between flooding-induced oxidative stress and antioxidant activity in the mangrove species *Kandelia candel*. Long periods of tidal immersion significantly inhibit the photosynthesis of mature leaves and increase the alcohol dehydrogenase and oxidase activity in roots of *K*. *candel*, which suggests that the roots are sensitive to anaerobiosis when in a waterlogged condition [16]. A study by Hoppe-Speer, Adams [35] on the response of *R*. *mangle* to salinity and inundation showed that the exposure to high salinity and continuous inundation induced a stress to the cultured seedlings as manifested by the frequent leaf shedding, excessive salt secretion and leaf necrosis. A study by Ye, Tam [37] showed that the species *B*. *gymnorrhiza* and *K*. *candel* have different responses to waterlogging stress dependent on the salinity and age of the seedlings. The activity of root oxidase significantly decreased in *B*. *gymnorrhiza* but increased in *K*. *candel* for less than 12 weeks of waterlogging treatment. However, in the leaves, the activities of both POD and SOD significantly increased in *K*. *candel*, whereas only POD increased in *B*. *gymnorrhiza*. A study field by He and Lai [44] on the critical level for forestation found that prolonged waterlogging induces increased superoxide dismutase (SOD) activity in roots, whereas moderate tidal flat inundation inhibits SOD activity in leaves.

### Integrating the ecophysiology into the mangrove rehabilitation scheme

Mangrove rehabilitation initiatives attract much attention from different sectors to foster mangrove recovery and biodiversity. The rehabilitation efforts received a renewed impetus after the 2004 Indonesia tsunami and again after the 2013 Typhoon Haiyan in the Philippines due to the highly valued mangrove ecosystem services specifically as a buffer and bioshield of coastlines [7, 45]. The results of the intensive efforts of mangrove rehabilitation programs are stories of mixed successes and failures. These efforts were often unsuccessful because of the high mortality of the planted seedlings due to inappropriate site selection [5, 8] and have failed to consider the species-specific ecophysiology. Most of the plantations were established in the lower intertidal zones using a single species of *Rhizophora* [6, 7] that are subjected to prolonged tidal inundation.

A study by Lewis III [46] reports that mangrove forest worldwide naturally exist in a raised and sloped platform above mean level, and inundated approximately 30%, or less of the tidal waters. More frequent flooding causes stress and eventually mortality. A study by He and Lai [44] along the Guangxi coast of China showed that the survival rate of *R*. *stylosa* sharply decreased from 88.9% to 44.0% as the tidal flat elevation decreased. In Sri Lanka, Kodikara, Mukherjee [47] reported that the survival of restoration projects ranged from 0 to 78%, and stressed that planting mangroves at the incorrect topography entails inappropriate soil conditions for growth. In laboratory condition, the photosynthetic and survival rates of *Avicennia marina*, *Heritiera littoralis* and *Bruguiera gymnorrhiza* decline with increasing salinity and submergence period [38]. A study by Mangora, Mtolera [38] stresses that submergence time and water salinity affect the sustainability of mangrove habitats and that the areas experiencing prolonged submergence with saline water might be the most severely affected. In the Philippines, there’s a widespread tendency to plant mangrove in areas that are not the natural habitat of mangroves particularly at the lower intertidal areas, thus resulted to low survival of 10 to 20% [5]. Of the few that survived, they had dismally stunted growth relative to the growth performance of those planted at the high intetidal areas [8].

Although mangroves are often described as being adapted to seawater flooding, they can cope with only limited frequency and duration of flooding; otherwise, growth, anatomical development, gas exchange, biomass partitioning, anti-stress enzymes and hormone levels are affected [17, 37, 48–50]. Climate change and sea-level rise are another threat that mangrove habitats are facing because of the challenges of increased flooding [51]. Mangrove seedlings, which are not yet sufficiently mature to withstand the environmental stressors, are most likely vulnerable to oxidative stresss and consequently leading to mortality. In extreme flooding conditions with prolonged submergence, the ability of the plant to respond to the stresses of submergence becomes crucial for its survival.

## Conclusions

This microcosm study mimicking the effect of water depth and tidal inundation showed that the species of *R*. *stylosa* had a low tolerance to inundation stress as shown by the induction of ROS and reduction of the photosynthetic pigments, carotenoids and Fv/Fm ratio. Inundation imposed a higher-degree of stress than that of the salinity effect; whereas continuous inundation caused sublethal damage as manifested by the chlorosis of the leaves. The biochemical stress responses of *R*. *stylosa* to salinity and inundation provided a new insight in understanding its ecophysiology and niche-width preference, which is a space or condition with less stress and deemed favorable for growth across the intertidal gradient. Extrapolating the optimum condition from both experiments and translating to field conditions, the frequently inundated areas, specifically at the seafront mangrove fringes subjected to longer hydroperiod inundation at spring tides, may suffer from oxidative stress, stunted growth and consequently poor survival. Knowledge of the species-specific ecophysiology of mangrove could provide a novel insight among the policymakers for future knowledge-based rehabilitation programs.

## Supporting information

S1 Table Biochemical responses of *R*. *stylosa* to salinity, water depth and inundation hydroperiod. (XLSX)

## References

[1] Butchart SH, Walpole M, Collen B, Van Strien A, Scharlemann JP, Almond RE, et al Global biodiversity: indicators of recent declines. Science. 2010:1187512. 10.1126/science.1187512 20430971

[2] Crossland CJ, Kremer HH, Lindeboom H, Crossland JIM, Le Tissier MD. Coastal fluxes in the Anthropocene: the land-ocean interactions in the coastal zone project of the International Geosphere-Biosphere Programme: Springer Science & Business Media, 232 p; 2005. 232 p.

[3] FAO. The world's mangroves 1980–2005. FAO (Food and Agriculture Organization) Forestry Paper 153, FAO, Rome. 2007.

[4] Richards DR, Friess DA. Rates and drivers of mangrove deforestation in Southeast Asia, 2000–2012. Proceedings of the National Academy of Sciences. 2016;113(2):344–9. 10.1073/pnas.1510272113 PMC4720307 26712025

[5] Primavera JH, Esteban JMA. A review of mangrove rehabilitation in the Philippines: successes, failures and future prospects. Wetlands Ecology and Management. 2008;16(5):345–58.

[6] Asaeda T, Barnuevo A, Sanjaya K, Fortes MD, Kanesaka Y, Wolanski E. Mangrove plantation over a limestone reef–Good for the ecology? Estuarine, Coastal and Shelf Science. 2016;173:57–64.

[7] Barnuevo A, Asaeda T, Sanjaya K, Kanesaka Y, Fortes M. Drawbacks of mangrove rehabilitation schemes: Lessons learned from the large-scale mangrove plantations. Estuarine, Coastal and Shelf Science. 2017;198:432–7.

[8] Samson MS, Rollon RN. Growth performance of planted mangroves in the Philippines: revisiting forest management strategies. AMBIO: A Journal of the Human Environment. 2008;37(4):234–40. 10.1579/0044-7447(2008)37[234:gpopmi]2.0.co;2 18686501

[9] Oh R, Friess D, Brown B. The role of surface elevation in the rehabilitation of abandoned aquaculture ponds to mangrove forests, Sulawesi, Indonesia. Ecological Engineering. 2017;100:325–34.

[10] Krauss KW, Lovelock CE, McKee KL, López-Hoffman L, Ewe SM, Sousa WP. Environmental drivers in mangrove establishment and early development: A review. Aquatic Botany. 2008;89(2):105–27.

[11] Friess D. JG Watson, Inundation Classes, and their Influence on Paradigms in Mangrove Forest Ecology. Wetlands. 2017;37(4):603–13.

[12] Watson JG. Mangrove forests of the Malay Peninsula. Malayan Forest Records. 1928;6:275.

[13] Ball MC. Ecophysiology of mangroves. Trees. 1988;2(3):129–42.

[14] Friess DA, Krauss KW, Horstman EM, Balke T, Bouma TJ, Galli D, et al Are all intertidal wetlands naturally created equal? Bottlenecks, thresholds and knowledge gaps to mangrove and saltmarsh ecosystems. Biological Reviews. 2012;87(2):346–66. 10.1111/j.1469-185X.2011.00198.x 21923637

[15] Naidoo G. Effects of waterlogging and salinity on plant-water relations and on the accumulation of solutes in three mangrove species. Aquatic Botany. 1985;22(2):133–43.

[16] Chen L, Wang W, Lin P. Photosynthetic and physiological responses of Kandelia candel L. Druce seedlings to duration of tidal immersion in artificial seawater. Environmental and Experimental Botany. 2005;54(3):256–66.

[17] Skelton NJ, Allaway WG. Oxygen and pressure changes measured in situ during flooding in roots of the Grey Mangrove Avicennia marina (Forssk.) Vierh. Aquatic Botany. 1996;54(2–3):165–75.

[18] He B, Lai T, Fan H, Wang W, Zheng H. Comparison of flooding-tolerance in four mangrove species in a diurnal tidal zone in the Beibu Gulf. Estuarine, Coastal and Shelf Science. 2007;74(1–2):254–62.

[19] Krauss KW, Twilley RR, Doyle TW, Gardiner ES. Leaf gas exchange characteristics of three neotropical mangrove species in response to varying hydroperiod. Tree Physiology. 2006;26(7):959–68. 16585041 10.1093/treephys/26.7.959

[20] McKee KL. Growth and physiological responses of neotropical mangrove seedlings to root zone hypoxia. Tree Physiology. 1996;16(11–12):883–9. 14871780 10.1093/treephys/16.11-12.883

[21] Ye Y, Tam NF-Y, Lu C-Y, Wong Y-S. Effects of salinity on germination, seedling growth and physiology of three salt-secreting mangrove species. Aquatic Botany. 2005;83(3):193–205.

[22] Wellburn AR. The spectral determination of chlorophylls a and b, as well as total carotenoids, using various solvents with spectrophotometers of different resolution. J Plant Physiol. 1994;144(3):307–13.

[23] DeEll JR, Toivonen PM. Use of chlorophyll fluorescence in postharvest quality assessments of fruits and vegetables Practical applications of Chlorophyll fluorescence in plant biology: Springer; 2003 p. 203–42.

[24] Jana S, Choudhuri MA. Glycolate metabolism of three submersed aquatic angiosperms during ageing. Aquatic Botany. 1982;12:345–54. 10.1104/pp.70.4.1125 PMC1065837 16662625

[25] Aebi H. Catalase in vitro. Methods Enzymol. 1984;105:121–6. 6727660 10.1016/s0076-6879(84)05016-3

[26] Nakano Y, Asada K. Hydrogen peroxide is scavenged by ascorbate-specific peroxidase in spinach chloroplasts. Plant Cell Physiol. 1981;22(5):867–80.

[27] MacAdam JW, Nelson CJ, Sharp RE. Peroxidase activity in the leaf elongation zone of tall fescue I. Spatial distribution of ionically bound peroxidase activity in genotypes differing in length of the elongation zone. Plant Physiol. 1992;99(3):872–8. 16669014 10.1104/pp.99.3.872 PMC1080558

[28] Lugo AE, Snedaker SC. The ecology of mangroves. Annual Review of Ecology and Systematics. 1974;5(1):39–64.

[29] Aziz I, Khan MA. Experimental assessment of salinity tolerance of *Ceriops tagal* seedlings and saplings from the Indus delta, Pakistan. Aquatic Botany. 2001;70(3):259–68.

[30] Jayatissa LP, Wickramasinghe W, Dahdouh‐Guebas F, Huxham M. Interspecific variations in responses of mangrove seedlings to two contrasting salinities. International Review of Hydrobiology. 2008;93(6):700–10.

[31] Chen Y, Ye Y. Effects of salinity and nutrient addition on mangrove Excoecaria agallocha. PloSOne. 2014;9(4):e93337. 10.1371/journal.pone.0093337 PMC3972235 24691495

[32] Clough B. Growth and salt balance of the mangroves Avicennia marina (Forsk.) Vierh. and Rhizophora stylosa Griff. in relation to salinity. Funct Plant Biol. 1984;11(5):419–30.

[33] Greenway H, Munns R. Mechanisms of salt tolerance in nonhalophytes. Annual Review of Plant Physiology. 1980;31(1):149–90.

[34] Wang W, Yan Z, You S, Zhang Y, Chen L, Lin G. Mangroves: obligate or facultative halophytes? A review. Trees. 2011;25(6):953–63.

[35] Hoppe-Speer SC, Adams JB, Rajkaran A, Bailey D. The response of the red mangrove *Rhizophora mucronata* Lam. to salinity and inundation in South Africa. Aquatic Botany. 2011;95(2):71–6.

[36] Pezeshki S, DeLaune R, Patrick W Jr. Differential response of selected mangroves to soil flooding and salinity: gas exchange and biomass partitioning. Can J For Res. 1990;20(7):869–74.

[37] Ye Y, Tam NF, Wong Y, Lu C. Growth and physiological responses of two mangrove species (Bruguiera gymnorrhiza and Kandelia candel) to waterlogging. Environmental and Experimental Botany. 2003;49(3):209–21.

[38] Mangora MM, Mtolera MS, Björk M. Photosynthetic responses to submergence in mangrove seedlings. Marine and Freshwater Research. 2014;65(6):497–504.

[39] Sharma P, Jha AB, Dubey RS, Pessarakli M. Reactive oxygen species, oxidative damage, and antioxidative defense mechanism in plants under stressful conditions. Journal of Botany. 2012;2012.

[40] Das SK, Patra JK, Thatoi H. Antioxidative response to abiotic and biotic stresses in mangrove plants: A review. International Review of Hydrobiology. 2016;101(1–2):3–19.

[41] Sugimoto M, Takeda K. Proteomic analysis of specific proteins in the root of salt-tolerant barley. Biosci Biotechnol Biochem. 2009;73(12):2762–5. 10.1271/bbb.90456 19966459

[42] García-Sánchez F, Syvertsen J, Gimeno V, Botía P, Perez‐Perez JG. Responses to flooding and drought stress by two citrus rootstock seedlings with different water‐use efficiency. Physiologia Plantarum. 2007;130(4):532–42.

[43] Wang H-m, Xiao X-r, Yang M-y, Gao Z-l, Zang J, Fu X-m, et al Effects of salt stress on antioxidant defense system in the root of Kandelia candel. Botanical Studies. 2014;55(1):57 10.1186/s40529-014-0057-3 28510976 PMC5430347

[44] He B, Lai T. Critical tidal level for forestation with hypocotyl of Rhizophora stylosa Griff along the Guangxi coast of China. Frontiers of Forestry in China. 2009;4(1):7–13. 17974232

[45] Wolanski E, Elliott M. Estuarine Ecohydrology: An Introduction: Elsevier; 2015.

[46] Lewis RR III. Ecological engineering for successful management and restoration of mangrove forests. Ecological Engineering. 2005;24(4):403–18.

[47] Kodikara KAS, Mukherjee N, Jayatissa LP, Dahdouh‐Guebas F, Koedam N. Have mangrove restoration projects worked? An in‐depth study in Sri Lanka. Restoration Ecology. 2017;25(5):705–16.

[48] Youssef T, Saenger P. Photosynthetic gas exchange and accumulation of phytotoxins in mangrove seedlings in response to soil physico-chemical characteristics associated with waterlogging. Tree Physiology. 1998;18(5):317–24. 12651371 10.1093/treephys/18.5.317

[49] Naidoo G, Rogalla H, Von Willert D, editors. Gas exchange responses of a mangrove species, Avicennia marina, to waterlogged and drained conditions. Asia-Pacific Conference on Science and Management of Coastal Environment; 1997: Springer.

[50] Chen L, Wang W, Lin P. Influence of water logging time on the growth of Kandelia candel seedlings. Acta Oceanol Sin. 2004;23:149–58.

[51] Ellison AM, Farnsworth EJ. Simulated sea level change alters anatomy, physiology, growth, and reproduction of red mangrove (Rhizophora mangle L.). Oecologia. 1997;112(4):435–46. 10.1007/s004420050330 28307619

